# Modified Pectoralis Major Tendon Transfer for Reanimation of Elbow Flexion as a Salvage Procedure in Complete Brachial Plexus Injury: A Case Report

**DOI:** 10.5704/MOJ.1303.004

**Published:** 2013-03

**Authors:** S Taran, SA Nawfar

**Affiliations:** Department of Orthopaedics, Universiti Sains Malaysia, Kubang Kerian, Malaysia; Department of Orthopaedics, Universiti Sains Malaysia, Kubang Kerian, Malaysia

## Abstract

Traumatic brachial plexus injuries rarely recover spontaneously and if the window period for neurotisation has elapsed, the only option for restoration of function lies in a salvage procedure. Many such salvage procedures have been described in the literature with variable functional results. We report the case of a 16-year-old boy who presented after unsuccessful treatment for a complete brachial plexus injury; we performed a pectoralis major tendon transfer to attain elbow flexion. Postoperatively, the elbow was splinted with flexion at 100°. After 4 weeks of immobilization the splint was removed and the patient could actively flex his elbow from 30° to 100°.

**Key Words:**

brachial plexus injury, salvage procedure, pectoralis major tendon transfer

## Introduction

The management algorithm for restoring elbow flexion in non-recovering post-traumatic brachial plexus injury (BPI) begins with neurosurgical procedures in the early stages (<6 months after injury) and salvage procedures thereafter^1^. These salvage procedures utilize either local or shoulder muscles for free muscle transfer^2^. One of the commonly used shoulder muscles for this procedure is the pectoralis major (PM) muscle. Previous use of the PM muscle can be broadly categorised into distal muscle transposition techniques and proximal tendon transfer techniques^3^. Here we report on a different technique that also utilises the PM muscle for reanimation of elbow function in a neglected post-traumatic BPI. This method is a modification of previously described techniques.

## Case Report

A 16-year-old Malay male was referred to our hand clinic for further management of a BPI to the right upper limb following a motor vehicle accident 3 months prior to presentation. Examination revealed a complete right BPI. He had normal motor power in the rhomboids and serratus anterior muscles. There was no evidence of scarring around the affected neck and shoulder region, nor clavicle or sternoclavicular joint disruption by clinical and radiological assessment. All the joints in the right upper limb were supple. We scheduled the patient for neurotisation of the right spinal accessory nerve (SAN) to the musculocutaneous nerve branch of the biceps (MBB) using a sural nerve graft. During surgery, stimulation of the MBB produced biceps contraction, indicating potential for nerve recovery; hence, the neurotisation was abandoned. We prescribed biceps brachii muscle re-education using electrical muscle stimulation to be administered by a physiotherapist.

**Fig. 1:**
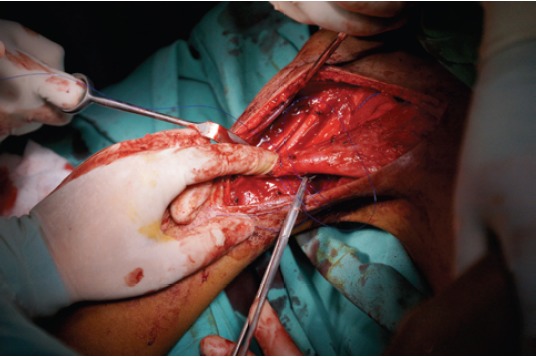
: Photograph depicting suturing of the pectoralis major tendon to the myotendinous junction of the biceps brachii to repair detachment from the humeral insertion.

Two months later (5 months post-injury), the patient was reviewed in the clinic and assessment revealed complete lack of biceps tone or active contraction and no other sensory or motor recovery. He was again scheduled for the neurotisation, but failed to present for the procedure. Instead, he presented for follow up 5 months later (10 months postinjury) and requested the previously planned neurotisation. We chose to abandon neurotisation in favour of muscle or tendon transfer as a salvage procedure to achieve elbow flexion. The PM muscle was assessed to be at Medical Research Council (MRC) grade 4, from MRC grade 0 at the previous examination, due to some recovery of his right pectoral nerves. His glenohumeral joint was relatively stable. We proceeded to perform a right-sided pectoralis major tendon transfer to attain elbow flexion at 11 months postbrachial plexus injury. Using a deltopectoral approach, the tendinous insertion of the PM was identified and detached. The musculotendinous unit was mobilised distally by dissecting the clavi-pectoral fascia without detaching either origin of the PM muscle. The PM tendon was subsequently sutured to the distal myotendinous junction of the biceps muscle using the Krackow technique to over-tensioning to 110° of elbow flexion and forearm supination (Figure 1). After wound closure, the elbow was splinted at 100° of flexion and forearm supination with a plaster-slab, collar and cuff.

The slab was maintained for 4 weeks during which isometric exercises were performed. At the 4 week follow-up appointment, the patient was able to actively flex at the right elbow from 30° to 100°, while attempting to adduct and flex his right shoulder; MRC grade was 3. Physiotherapy assisted passive range of motion (ROM) exercises were commenced and graduated to active ROM exercises 2 weeks later. Once painless active ROM was possible, rehabilitation focussed on muscle strengthening to improve the MRC grade.

## Discussion

The aim of late reconstruction is to restore elbow flexion using a tendon or muscle transfer procedure to restore strength and functional ROM to 30-130° without excessive pronation. Previously described surgical options include the Steindler procedure, latissimus major muscle transfer, pectoralis major muscle transfer, pectoralis minor muscle transfer, triceps muscle transfer, sternocleidomastoid muscle transfer and last but not least free muscle transfer (e.g., gracilis muscle transfer)^4^.

The primary factor guiding our choice of procedure was the type and extent of the BPI. In the present case, this limited our options to use of a muscle group with motor power of at least grade 4 MRC or higher. Other considerations included muscle excursion, alignment, cosmesis and preoperative ROM. For the current case, the only ideal donor was the PM muscle. As described by Heirner et al., there are 2 scenarios in which PM muscle transfer techniques should be used. The first being the distal muscle transposition technique, where the PM origin is transposed and tenodesis is performed to the biceps brachii insertion. This category can be further divided into unipolar or bipolar and partial or complete distal transfer. The second, a proximal tendon transfer technique, where the insertion of the PM tendon to the humerus is detached and tenodesis to the biceps brachii tendon insertion is accomplished using an interposing tensor fascia lata graft. This technique also involves detachment of the clavicular origin of the PM to gain excursion^3^.

Our technique is a modification of the latter. We adopted this technique due to our intra-operative findings, revealing that the sternocostal portion of the PM was atrophied. This enabled the clavicular portion to be mobilised with sufficient excursion without detachment. This technique avoids the need for harvesting a tendon graft and accompanying donor side morbidity. It also reduces the amount of required dissection as there was no need for detachment from the PM muscle origin or exposure of the radial tuberosity (insertion of biceps brachii). The major disadvantage though was the bowstringing effect subcutaneously, which was cosmetically unfavourable.

Once soft tissue healing is optimal, we plan to reassess the patient’s shoulder stability on the affected side as the previous dynamic stabilization by the PM was sacrificed. If stability is compromised, shoulder fusion will be offered as an option to the patient. This would further enhance the strength of elbow flexion. The long-term plan is to reestablish finger and wrist flexion using a gracilis free muscle transfer, provided the patient first achieves stable shoulder and functional elbow flexion.

## Conclusion

Salvage procedures for post-traumatic complete brachial plexus injuries are performed with the aim of restoring some function to a limb that has none. The surgical priority is to reestablish functional elbow flexion first^5^. We used a modification of existing pectoralis major muscle transfer techniques that offers a new alternative for those who give precedence to function over cosmesis. As this was only our first such case and only a series of cases will enable us to sufficiently assess outcomes for this technique and its ideal rehabilitative protocol.
